# Extensive ecchymosis and retroperitoneal hemorrhage due to acquired hemophilia-A following influenza vaccination: A case report

**DOI:** 10.1097/MD.0000000000038300

**Published:** 2024-05-24

**Authors:** Chi Sheuan Chin, Shinn-Jye Liang

**Affiliations:** a Department of Internal Medicine, China Medical University Hospital, China Medical University Hospital, Taichung City, Taiwan; b Division of Pulmonary and Critical Care, Department of Internal Medicine, China Medical University Hospital, Taichung City, Taiwan.

**Keywords:** acquired hemophilia-A, extensive ecchymosis, influenza vaccination, retroperitoneal hemorrhage

## Abstract

**Introduction:**

Acquired hemophilia-A (AHA) is a rare but potentially life-threatening impaired coagulation disorder characterized by the development of autoantibodies against clotting factor VIII. Only a few case reports have been experienced with influenza vaccine-triggered AHA. Here, we report a case of severe hemorrhagic disorder due to AHA following influenza vaccine, which was successfully treated.

**Patient concerns:**

The patient presented to the emergency department because of several severe, progressively worsening bruises after receiving the influenza vaccination. Consequently, the patient required intubation due to nasal-oral bleeding, which compromised the airway, and retroperitoneal hemorrhage with shock also developed.

**Diagnosis:**

AHA was confirmed through a coagulation factor assay, including coagulation activity and antibody testing, which is possibly triggered by influenza vaccination.

**Intervention:**

Low-dose cyclophosphamide and hydrocortisone were prescribed until activated partial thromboplastin time showed normal levels. Coagulation factor VIIa was administered, and aggressive blood transfusion was carried out concurrently to address the blood loss.

**Outcomes:**

The upper airway bleeding subsided and bleeding tendencies had been corrected to normal. The patient was smoothly weaned from the ventilator and recovered from critical illness. She was then discharged on the 19th day.

**Lessons:**

The activated partial thromboplastin time mixing test can be performed immediately to establish the initial differential diagnosis and treatment plan for severe coagulopathy. AHA may be triggered by vaccination, with the hypothesis of activation of autoantibodies and molecular mimicry; this mechanism should be further studied.

## 1. Introduction

Acquired hemophilia-A (AHA) is a rare disease characterized by hemorrhagic disorder wherein autoantibodies develop against factor VIII. Whenever there is bleeding in a patient who does not have any history of bleeding and has an unexplained prolonged activated partial thromboplastin time (aPTT), hemophilia should be kept in mind. The essential underlying causes are not clearly known. Some reported risk factors for AHA include medication, autoimmune diseases, pregnancy, or malignancy. Controlling bleeding and eradicating autoantibodies against clotting factors are very challenging for primary care physicians. Here, we present a case with AHA following influenza vaccination. The patient initially presented with injection site bruise, which then progressed to extensive upper limb ecchymosis, ultimately leading to hypovolemic shock due to retroperitoneal hemorrhage. This is a rare and life-threatening case of influenza vaccination-related AHA. To date, only a few cases have been reported in the past two decades.

## 2. Case presentation

An 80-year-old female without any underlying disease presented to the emergency department on November 4, 2022, with progressive right arm ecchymosis that persisted for 2 weeks. She had received an influenza vaccination [A/Singapore/Infimh-16-0019/2016 (H3N2)-Like Virus; A/Michigan/45/2015 (H1N1)Pdm09-Like Virus ADIMFLU-S (QIS)] on October 18, 2022, with intramuscular injection being smooth and without any complications. However, she noticed the development of ecchymosis on her right upper arm three days after receiving the influenza vaccine. Initially, the bruises measured 0.5 cm in size and gradually increased in size, eventually spreading to her entire arm over the course of 1 week.

**Figure 1.**
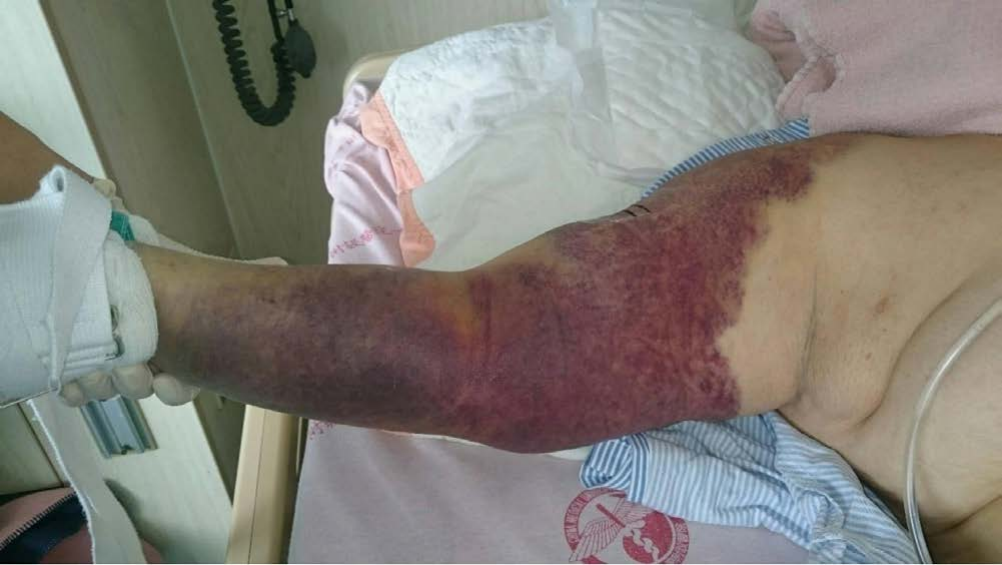
Severe bruising was on the right arm and forearm upon presentation.

**Figure 2.**
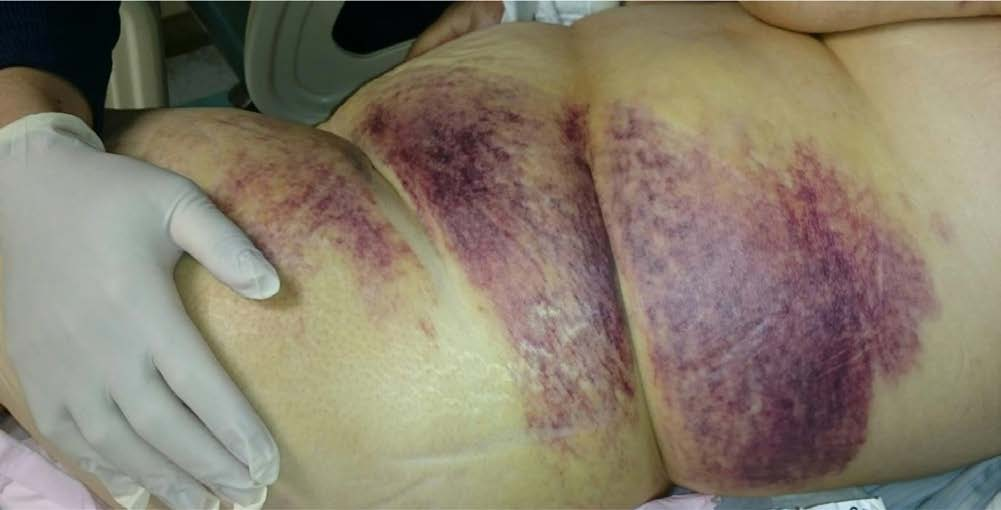
Ecchymosis on the left flank closely resembled Grey-Turner’s sign.

The initial physical examination revealed a blood pressure of 102/69 mm Hg, a heart rate of 105/min, multiple bruises (Figs. 1 and 2), and evidence of nasal-oral bleeding. While conducting the examination, a substantial blood clot was observed in the nasal-oral region, accompanied by alteration in mental status. Consequently, emergency intubation was promptly carried out to ensure unobstructed airway protection. Subsequently, the patient was transferred to the intensive care unit for comprehensive treatment.

**Figure 3.**
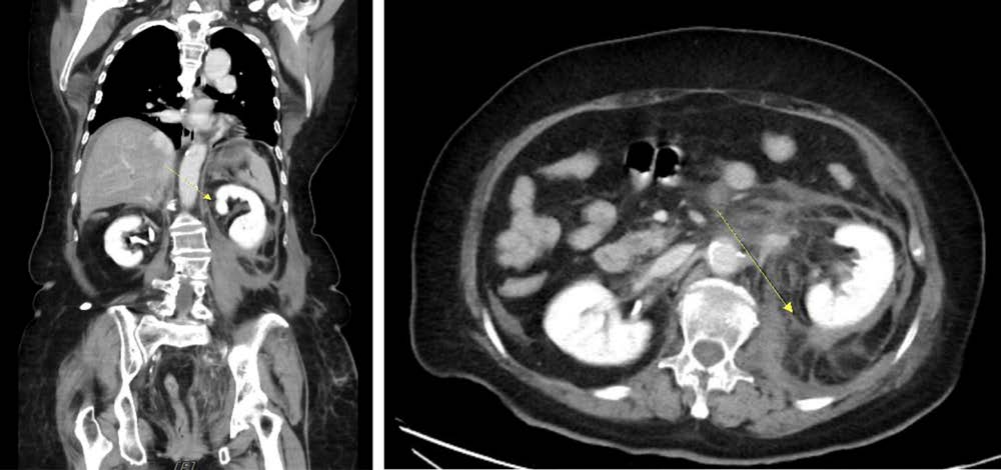
Abdominal CT scans revealed a left retroperitoneal hematoma, as indicated by the left arrow in both the axial and coronal views, respectively. CT = computed tomography.

Among the laboratory findings, normocytic anemia with hemoglobin level of 6.7 g/dL was observed, along with coagulopathy indicated by prothrombin time of 15.3 seconds, aPTT of 78.7 seconds, and a platelet count of 102,000/μL. A mixing study was conducted to differentiate between coagulation factor deficiency and the presence of a coagulation factor inhibitor. In this case, the aPTT test remained prolonged at 60 seconds, even after 2 hours of incubation. An abdominal computed tomography was performed, revealing a left retroperitoneal hematoma (as shown in Fig. 3) consistent with left flank Grey-Turner sign.

Hemophilia was suspected, and meanwhile, controlling and preventing massive blood loss was deemed crucial. Eight units of leukocyte-reduced packed red blood cells, 12 units of platelet concentrate, and 4 units of fresh frozen plasma were administered, with close monitoring of hemoglobin, platelets, prothrombin time, and aPTT. Additionally, Eptacog alfa (Factor 7) 50 KIU/vial was prescribed every 3 hours, along with hydrocortisone 60 mg once a day. After 6 days of treatment, the results of the coagulation factor and inhibitor assay were released, showing Factor 8 assay: 1%, Factor 9 assay: 60.5%, Factor 8 inhibitor: 7.0 BU, and Factor 9 inhibitor was negative.

A diagnosis of AHA was made, and low-dose chemotherapy with intravenous cyclophosphamide at a daily dose of 100 mg was initiated. On the seventh day of treatment, aPTT became normalized to 37.7 seconds. Subsequently, the intravenous form of cyclophosphamide was switched to an oral form at a dose of 50 mg once a day. Nasal and oral bleeding subsided, and bruising improved. She was smoothly weaned off the treatment and transferred to the general ward on the 14th day. Eventually, she was discharged on day 19th of hospitalization.

## 3. Discussion

Whenever a bleeding disorder is suspected, it is crucial to consider the underlying pathogenesis, including plasma protein defects, platelet abnormalities, and defects in platelet–endothelial cell interactions.^[1]^ The mechanism behind influenza vaccination-triggered AHA remains unclear, but it was strongly suspected to be related to an autoimmune disorder.^[2]^ The incidence of AHA is approximately 1.5 cases per million per year and is characterized by autoantibodies directed against circulating coagulation factor (F) VIII.^[3,4]^ If the aPTT is prolonged, a mixing aPTT test should be performed.^[3,4]^

It is important to note that the severity of bleeding upon initial presentation does not necessarily predict the recurrence or subsequent severity of bleeding. The reported mortality rate ranges between 8% and 22%, with spontaneous bleeding being the primary cause of mortality.^[1,5]^ Therefore, close monitoring of laboratory data and vital sign is essential, and invasive procedures should be avoided whenever possible. However, in cases when urgent procedures are necessary, hemostatic therapy should be administered both and after procedures.^[5]^ The most common presentation of AHA is subcutaneous hematomas (80%), which can occur in various locations, including intramuscular (45%), gastrointestinal (21%), genitourinary (9%), and retroperitoneal (9%).^[6]^

Immediate eradication of inhibitory autoantibodies is imperative, regardless of the severity of bleeding.^[7]^ The current recommendation is to use corticosteroids in combination with cyclophosphamide. In cases where the patient does not respond or cannot tolerate the side effects of the first-line treatment, alternative options such as anti-CD20 monoclonal antibodies and calcineurin inhibitors may be considered.^[1,8,9]^ It is worth nothing that high-dose intravenous immunoglobulin, either alone or in combination with other immune-suppressants is not recommended for the treatment of AHA.^[8,9]^

Although we successfully identified and treated the patient effectively, there are limitations to our report. It is difficult to confirm a definitive link between influenza vaccination and AHA. A precise diagnostic tool to pinpoint AHA caused by vaccines was not available; we lack direct molecular evidence. Clinically, we can only use history taking to collect information for diagnosis and rule-out approach. In the future, with thorough studies on AHA with a molecular biology basis, perhaps within one serum examination, we can avoid vaccinating individuals who are not suitable for the influenza vaccine.

## 4. Conclusion

AHA is a potentially life-threating condition characterized by severe bleeding. Patients presenting with an isolated prolongation of aPTT should undergo additional mixing tests. The diagnosis of acquired hemophilia A hinges on the detection of inhibitors against factor VIII. Maintaining hemostasis and eradicating inhibitors should proceed simultaneously and rapidly. It is worth noting that there is currently a lack of sufficient data and studies regarding the triggering of acquired hemophilia A by influenza vaccination.

## Author contributions

**Conceptualization:** Chi Sheuan Chin.

**Investigation:** Chi Sheuan Chin.

**Project administration:** Chi Sheuan Chin.

**Supervision:** Shinn-Jye Liang.

**Writing—review & editing:** Shinn-Jye Liang.
